# Talk like me: Exploring the feedback speech rate regulation strategy of the voice user interface for elderly people

**DOI:** 10.3389/fpsyg.2023.1119355

**Published:** 2023-03-17

**Authors:** Junfeng Wang, Shuyu Yang, Zhiyu Xu

**Affiliations:** College of Design and Innovation, Shenzhen Technology University, Shenzhen, China

**Keywords:** voice user interface (VUI), elderly people, feedback speech rate, regulation strategy, speech convergence

## Abstract

Voice user interface (VUI) is widely used in developing intelligent products due to its low learning cost. However, most of such products do not consider the cognitive and language ability of elderly people, which leads to low interaction efficiency, poor user experience, and unfriendliness to them. Firstly, the paper analyzes the factors which influence the voice interaction behavior of elderly people: speech rate of elderly people, dialog task type, and feedback word count. And then, the voice interaction simulation experiment was designed based on the wizard of Oz testing method. Thirty subjects (*M* = 61.86 years old, SD = 7.16; 15 males and 15 females) were invited to interact with the prototype of a voice robot through three kinds of dialog tasks and six configurations of the feedback speech rate. Elderly people’s speech rates at which they speak to a person and to a voice robot, the feedback speech rates they expected for three dialog tasks were collected. The correlation between subjects’ speech rate and the expected feedback speech rate, the influence of dialog task type, and feedback word count on elderly people’s expected feedback speech rate were analyzed. The results show that elderly people speak to a voice robot with a lower speech rate than they speak to a person, and they expected the robot feedback speech rate to be lower than the rate they speak to the robot. There is a positive correlation between subjects’ speech rate and the expected speech rate, which implies that elderly people with faster speech rates expected a faster feedback speech rate. There is no significant difference between the elderly people’s expected speech rate for non-goal-oriented and goal-oriented dialog tasks. Meanwhile, a negative correlation between the feedback word count and the expected feedback speech rate is found. This study extends the knowledge boundaries of VUI design by investigating the influencing factors of voice interaction between elderly people and VUI. These results also provide practical implications for developing suitable VUI for elderly people, especially for regulating the feedback speech rate of VUI.

## 1. Introduction

Research in speech recognition began in the 1950s. Early technology only recognizes the English pronunciation of 10 numerals (Li, 2018; Shahrebaki et al., 2018). Current voice interaction systems have been employed in many devices that feature human-computer interaction technology. It can recognize complete human natural language utterances, forming a significant and booming market segment (Zen et al., 2013). In the process of voice interaction, the user activates the device by speaking specific voice commands. After receiving the commands, the device recognizes the commands and gives feedback. The feedback is then transformed into a sound that simulates a human voice with artificial speech synthesis technology and plays through the speaker to form voice feedback (Hess and Zellman, 2018; Singh et al., 2021).

The visual interaction interface necessitates learning its operation (Page, 2014), understanding the meaning of graphical elements, and searching for the object to be operated within the visual range, all of which lead to high learning costs (Liu and Ma, 2010; Bai et al., 2020). Voice interaction, in contrast, only requires users’ short-term memory and clear verbal expression, with low learning costs. Thus, it is appropriate for children, the elderly, and people with visual impairment (Huang and Liu, 2017; Zhang and Zhang, 2019; Pradhan et al., 2020; Hua, 2021; Guo et al., 2022). Some scholars have applied it to designing and developing products for the elderly. For instance, Antonio et al. (2014) built an intelligent system with multichannel interaction for the elderly by collecting a language database of them and constructing an elderly-specific language recognizer. Jia (2018) researched the elderly using the SJTU user research system and constructed corresponding voice interaction application scenarios for them. Furthermore, they concluded that when completing task-oriented dialogs, short and simple dialogs can lessen the memory load of the elderly and increase task completion, according to the experiment.

However, the design of current voice interaction systems has not considered the reduced hearing, cognitive and comprehension abilities of the elderly, let alone specific adjustments to the feedback speech rate of the system (Murad et al., 2019). This causes the issue that older users frequently have difficulty hearing or remembering while using voice interaction devices (Czaja et al., 2006; Lee and Coughlin, 2015), which significantly negatively impacts their interaction effectiveness and user experience (Baba et al., 2004; Lee, 2015; Zhang, 2021). This paper takes the feedback speech rate of voice interaction systems for the elderly as the research object. The feedback speech rate that the elderly expect in different task scenarios is collected through voice interaction simulation experiments. The speech rate regulation strategy of the voice interaction system is constructed accordingly to improve the efficiency and user experience of the elderly when using the system.

## 2. Literature review

### 2.1. Speech rate of the elderly

Communication is a process in which both the speaker and the addressee express information by language, and its purpose is to convey information. Speech accommodation theory (SCT) is a sociolinguistic theory developed recently (Yuan, 1992; Ma, 1998). The theory suggests that the addressee’s speech acts characteristics can be a reference standard for the speaker to regulate speech acts. Speech convergence is a phenomenon that in daily conversation, the speaker’s speech pattern (diction, speech rate, grammar, phonology, et al.) is influenced by the addressee’s speech pattern and adjusted to it to gain the addressee’s approval and affirmation (Beebe and Giles, 1984).

Speech convergence is more likely to occur when the speaker is a subordinate or junior, and the conversation is serious. In this situation, the speaker’s speech pattern will converge with those of the addressee (Brennan and Clark, 1996; Barr and Keysar, 2002; Gijssels et al., 2016). Kemper’s study found that when young people converse with the elderly, they will slow their speech rate and reduce the length and complexity of the discourse to help the elderly understand the message, i.e., speech regulation mechanisms toward the elderly (Kemper, 1994). A study by Jiang (2017) noted that young people adopt speech convergence in telephone conversations with aged people, including but not limited to speech rate, average sentence length, and pause duration. The aim is to assist aged people in understanding the message, gain their trust, and enhance emotional intimacy.

Human-computer voice interaction design aims to simulate human-to-human verbal communication (Murad et al., 2019). Many intelligent voice devices define the voice interaction system as the user’s “intelligent voice assistant” (Rakotomalala et al., 2021) and refer to the user as the “master” during the conversation. This design reflects the relationship of ownership, subordination, and domination between the user and the voice device, comparable to the relationship between superiors and subordinates in interpersonal interactions (Nass et al., 1994; Powers and Kiesler, 2006; Ostrowski et al., 2022). On the other hand, voice interaction devices are inanimate objects, and users have lower intimacy and trust during initial use. They also interact more cautiously, which is not conducive to conveying information effectively (Dautenhahn, 2004; Höflich and El Bayed, 2015; Song et al., 2022). Thus, it is reasonable and essential to apply speech convergence strategies to improve the dialog relationship between voice interaction devices and users.

Based on speech accommodation theory, when elderly people interact with the system through voice, the system adjusts speech acts characteristics of the feedback following the elderly’s, which will enhance the emotional experience of the elderly (Myers et al., 2018, 2019). The paper analyzes the correlation between the speech rate of the elderly and their expected feedback speech rate. It also summarizes the feedback speech rate regulation strategies of the voice interaction system toward elderly people based on experimental results.

### 2.2. Dialog task type

In existing research, the types of dialog tasks were divided into non-task-oriented dialogs and task-oriented dialogs according to the different purposes (Luna-Garcia et al., 2018) for which users use voice interaction devices (Chen et al., 2017).

Non-task-oriented dialogs primarily refer to forms of interaction in which users have no clear expectations or specific goals about the feedback from the voice interaction system. Typical applications include listening to music, stories, and operas. The device recognizes the type of content users want to listen to after activating the device with the wake-up word. Then the device starts to play the audio, and users enter the listening stage. Users do not initiate the voice interaction process again until the audio finishes or the audio is dissatisfying. In this usage scenario, the user’s purpose is to pass the time and relieve loneliness. When interacting with devices, users can identify only some of the information intentionally or memorize it. Users only must confirm that the device is playing the needed content, which requires a low cognitive load.

Task-oriented dialogs mainly refer to voice interaction systems that assist users in accomplishing specific tasks, such as checking the weather, making hotel or restaurant reservations, et al., by single or multiple rounds of dialogs. With a specific goal, users activate the device and give the corresponding voice command when utilizing such functions. Then users extract the information needed from the feedback played by the device and store it in short-term or long-term memory, which can be applied to the specific task. A more complex situation is that the voice interaction system conveys information to users through multi-round dialogs, such as multi-round quizzes and quiz games. In these dialogs, users must mobilize brain functions such as thinking and memory to participate in the interaction. They must also comprehend and analyze the received feedback to respond with the highest cognitive load (Sayago et al., 2019; Moore and Urakami, 2022).

As shown in Table 1, this paper conducts a voice interaction simulation experiment based on the two kinds of dialog tasks to analyze the influence of dialog task types on the expected feedback speech rate for the elderly. However, people interact with VUI with no clear goals, they also execute a dialog task. It is a little bit confusing to name this kind of interaction as a non-task-oriented dialog. So, goal-oriented, and non-goal oriented are used to describe the interactions that with and without a clear goal, respectively.

**Table 1:** Functions and dialog tasks classifications of voice interaction devices.

Types of dialogue task	Examples of device functions	Interaction behavior characteristics
Non-goal-oriented	Play music, stories, operas, etc.	Users can recognize only some feedback information, primarily for pleasure with poor purpose.
Goal-oriented	The broadcast, weather forecast, news, English words, etc.	Dialogs with single or more rounds, users have strong purposiveness, must recognize, and memorize the feedback information from the system, and sometimes respond to the feedback.

### 2.3. Word count of single feedback

Human brain nerve cells gradually diminish after the age of 50 (Svennerholm et al., 1997; Scahill et al., 2003). Although the brain’s basic functions can generally be maintained, the volume of brain tissue may atrophy to varying degrees in some individuals, resulting in memory loss and personality changes. Brain nerve cells also impact the regulatory role of the brain’s involvement in other organs, affecting other organs’ functional performance (Wang, 2002). Compared to young people, the perceptual abilities of the elderly become blunted, and degenerative changes may occur in vision, visual perception, hearing, and auditory perception (Hawthorn, 2000; Yuan et al., 2006; Wilkinson and Cornish, 2018).

Elderly people find it more challenging to accept new things (Ziman and Walsh, 2018; Kowalski et al., 2020) and technologies (Kalimullah and Sushmitha, 2017) and acquire external information due to the deterioration in their perceptual abilities. Therefore, voice interaction simulation experiments were designed based on the feedback of different word counts from the voice interaction system to investigate the impact of the word count in single feedback on the elderly’s expected feedback speech rate.

### 2.4. Voice user interface for elderly people

Voice activated human machine interaction has developed rapidly in recent years. This has attracted extensive attention from the academic community. VUIs offer elderly people multiple advantages over traditional GUI/hardware interfaces by being fewer motor skills required, efficient of getting information, intuitive to interact, and rich of meaning through tone, volume, intonation, and speed (Sayago et al., 2019). More researchers are involved in the research of VUI for elderly people.

Ziman and Walsh (2018) studied the factors affecting seniors’ perceptions of VUI, and indicated that familiarity, usability, habit, aversion to typing, and efficiency of voice input are the most critical factors that influenced the seniors’ perceptions and acceptance of VUI. Research (Pradhan et al., 2020; Song et al., 2022) focused on the adoption and usage of VUI by elderly people with low technology use found that perceived usefulness, perceived ease of use, and trust are decisive factors that determined elderly people adapt VUI or not and influenced their attitudes to VUI. After studied the patterns of tactics that people employ to overcome the problems when interacting with VUI, Myers (Myers et al., 2018) indicated that feedback strategy could be a worthy point to improve VUI’s user experience.

Meena et al. (2014) proposed a data-driven approach to building models for online detection of suitable feedback response locations in the user’s speech. The results from the user evaluation through human computer interaction show that the model trained on speaker behavioral cues offers both smoother turn-transitions and more responsive system behavior. Other research that concentrated on feedback position during conversation identified feedback locations through multimodal models (Boudin et al., 2021), Interdisciplinary Corpus (Boudin, 2022), and voice activity (Truong et al., 2010; Ekstedt and Skantze, 2022).

## 3. Methods

This study explores the influence of dialog type and feedback word count on the user’s expected feedback speech rate.

### 3.1. Research design

This study applies Wizard of Oz testing to conduct voice interaction simulation experiments (Cordasco et al., 2014; Ma et al., 2022). Wizard of Oz testing is a method in which the tester acts as a “wizard” to manipulate the object to be tested to make it interact with the subject and collects relevant experimental data (Dahlbäck et al., 1993). This method is widely applied to study the usability and user acceptance of voice interaction systems, natural language applications, command languages, imaging systems, and pervasive computing applications in the prototype stage (Shin et al., 2019). In the development of voice-interactive product design, Wizard of Oz can assist UX researchers in cutting costs by quickly testing the usability of products at different stages (White and Lutter, 2003). Some studies used the Wizard of Oz testing to investigate the elderly’s acceptance of smart home products equipped with voice-interactive systems (Portet et al., 2013; Porcheron et al., 2020).

Before the experiment, dialog tasks between users and voice interaction devices were designed based on the current mainstream voice interaction process. During the experiment, subjects are first informed about the experiment’s background and the task requirements. Then subjects perform the appropriate voice interaction behavior in line with the task requirements, and the experimenter manipulates the product to provide feedback. The assistant collected subjects’ speech rate data during the process and the subjects’ expected feedback speech rate with the speech rate satisfaction scale (Ghosh et al., 2018). The assistant also needs to observe and record subjects’ performance and attitudes. Finally, user interviews and quantitative analysis were conducted to explore the users’ expectations of the feedback speech rate under different task scenarios (Iniguez et al., 2021).

### 3.2. Subjects and settings

To select the qualified subjects, the experimenter explained the process and conducted a pre-talk test to ensure that the subjects could hear the conversation clearly in the experimental environment and understand the overall process before the experiment. Thirty elderly people with normal hearing and no obvious cognitive impairment were chosen for the study; 10 were between 60 and 70 years old, six were 70 years old or above, and the others were 50 and 60.

**Figure 1:**
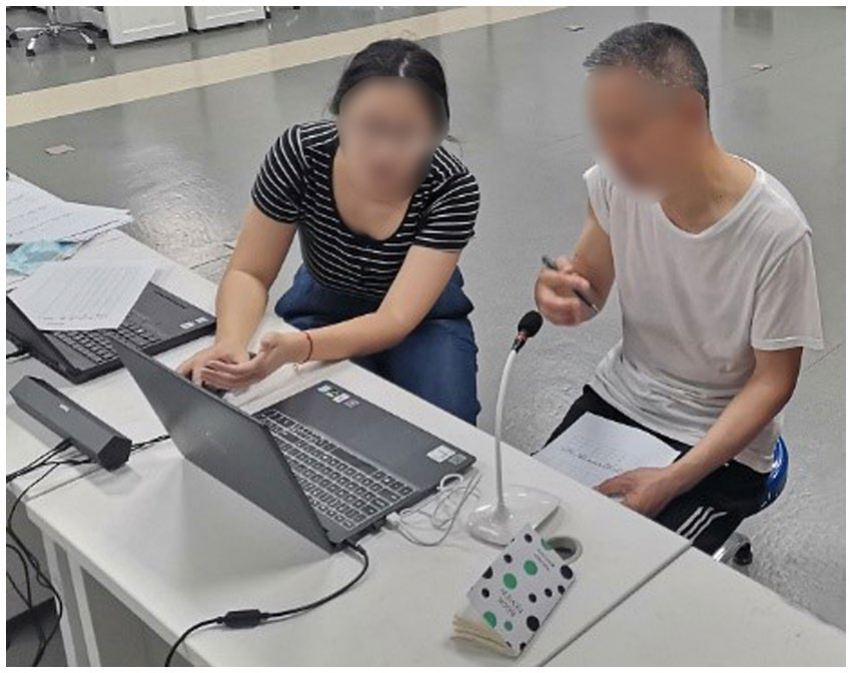
Environment for voice interaction simulation.

The experiment was conducted in a quiet indoor environment with soft light (Figure 1). The experimenter had previously explained the experimental task process to the subjects before the experiment. The subjects sat facing the 15.6-inch display, a Philips SPA510 dual-channel speaker was used to play the voice interaction content, and a Newman ZM02 microphone collected the user’s voice commands.

### 3.3. Materials

#### 3.3.1. Dialog tasks

Currently, intelligent voice interaction devices are widely used in daily life. The cognitive load of the elderly is low when engaging in non-goal-oriented conversations while high when engaging in goal-oriented conversations.

To investigate the correlation between the user’s expected feedback speech rate and the type of dialog task, Task 1 is set as a non-goal-oriented dialog in the scenario that the elderly was bored at home and wanted to listen to the radio for entertainment. It is a light-load interaction task. Task 2 is a goal-oriented dialog, with the scenario of elderly people checking the weather the next day before going out, which was a heavy-load interaction task. In order to find the relationship between the user’s expected feedback speech rate and the word count in single feedback, Task 3 is also designed as a goal-oriented dialog in which the scenario is that the elderly is checking the next day’s schedule. The word count in the feedback of Task 3 is different from that in Task 2.

In a text-to-speech system, the length of punctuation that pauses in the corpus is approximately equal to a word. So, the punctuation in the corpus is counted into the total words. Table 2 shows the three dialog tasks. The voice dialog is in Chinese, so the feedback words of the three tasks are also counted in Chinese characters.

**Table 2:** Dialog task of voice interaction experiment.

No.	Task	Words count	Type of dialog task	Dialog scenario
Task 1	Listen to the news	37	Non-goal-oriented	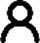 What is in the news today?
				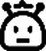 International rating agency Fitch downgraded the credit ratings of 33 economic entities in the first half of the year due to the epidemic, which surged to a record high.
Task 2	Check the weather	37	Goal-oriented	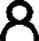 How is the weather in Shenzhen tomorrow?
				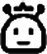 There will be light rain in Shenzhen tomorrow; the temperature is 28–22 Celsius, which is suitable for wearing short sleeves or shirts. Please remember to bring an umbrella when you go out.
Task 3	Check the schedule	18		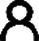 What is the time of the physical examination tomorrow morning?
				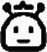 Okay, your appointment for a physical examination is at 9:30 tomorrow morning.

To avoid sequential effects, a set of experiments was conducted with dialogs tasks in three scenarios, including Task 1, “listen to the news,” Task 2, “check the weather,” and Task 3, “check the schedule.” Each subject was required to complete six experiments with six random gears of feedback speed for all tasks.

#### 3.3.2. Feedback speech rate

Speech rate typically refers to the speed of articulation, while it can also refer to the auditory perceptual impression of the pacing of words (Cao, 2003). The acceptable speech rate is below 300 words/min, and over this range, listeners may have difficulty following the conversation (Portet et al., 2013). Besides, a speech rate of 100–150 syllables/min is considered a “super-slow speech rate,” which is rare in daily conversations (Meng, 2006). Therefore, six speech rates were defined as the feedback speed configurations for the experiment, as shown in Table 3. The six feedback speech rates of the system were 2.25 words/s (135 words/min), 2.75 words/s (165 words/min), 3.25 words/s (195 words/min), 3.75 words/s (225 words/min), 4.25 words/s (255 words/min) and 4.75 words/s (285 words/min).

**Table 3:** Configurations of feedback speech rate.

No.	1	2	3	4	5	6
Feedback speech rate(words/s)	2.25	2.75	3.25	3.75	4.25	4.75

#### 3.3.3. Feedback corpus

**Figure 2:**
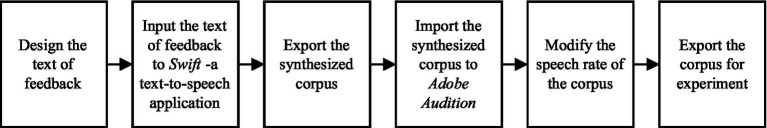
The process of synthesizing the feedback corpus.

The text of the feedback corpus was synthesized into speech using the Swift text-to-speech system, and the pronunciation source was the built-in standard female voice with a 16 khz sampling rate. Figure 2 depicts the process of the feedback corpus synthesizing. Firstly, the text of the feedback corpus was designed according to the purpose of the dialog. Secondly, each speech was generated by Swift, the text-to-speech system. Thirdly the synthesized speech was imported into Adobe Audition to modify the speech rate according to the configurations in Table 3, and then export the corpus for the experiment.

### 3.4. Experimental process

**Figure 3:**
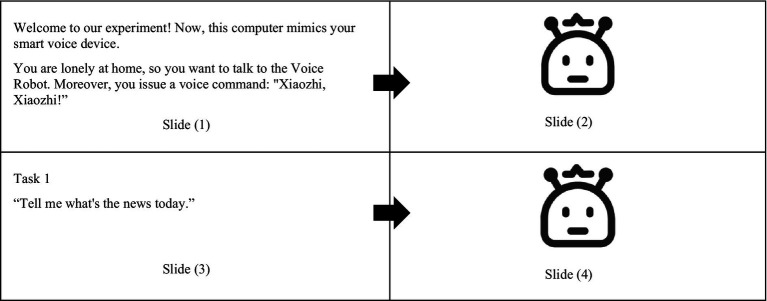
The Prototype of the voice interaction system.

The experiment was designed based on Wizard of Oz testing, in which the subject initiated the voice interaction, and the experimenter acted as a “wizard” to operate the prototype of the voice interaction system to give feedback. The prototype was made of an avatar and synthesized feedback corpus. To guide subjects, we made some slides incorporated with the prototype (see Figure 3). The dialog between subjects and voice robot prototype was simulated by switching the slides.

Take Task 1, “listen to the news,” as an example. The experimenter explained the experimental process to the subject before it started. To ensure the experiment ran smoothly, the subjects were informed of the simulated scenario of the dialog task and activated the voice interaction system in the pre-test. Slide (1) is the experiment guide, showing the subjects the dialog scenario and wake-up words. After issuing the wake-up word, subjects entered slide (2), and the experimenter played feedback, “Hey, I’m here!” indicating that the system was activated. The slide (3), an experimental guide, was shown to suggest to the subjects the dialog scenarios and voice commands to be issued. During the experiment, to enhance the subjects’ immersion, waiting and speaking animations were added to the icons of the robot.

The experiment was conducted with a 5-point Likert scale to evaluate subjects’ satisfaction with the feedback speech rate of the system (Feng, 2002). Subjects’ satisfaction was calculated based on the options corresponding to the ratings in Table 4. A higher satisfaction score of the feedback speech rate indicates a higher acceptance by the user.

**Table 4:** Speech rate satisfaction scale.

Options	Description	Rating
1	The system speaks too slowly, and I cannot accept it.	1 point
2	The system speaks slowly, but I can accept it.	3 points
3	The system speaks at just the right speed.	5 points
4	The system speaks fast, but I can accept it.	3 points
5	The system speaks too fast, and I cannot accept it.	1 point

While performing the experimental task, the subjects’ speech content was recorded with Adobe Audition CC2020 and a microphone in 16 kHz, mono, 32-bit audio format.

When subjects finished all dialog task, some open-ended questions were asked. The mainly purpose of this post-experiment interview is to know the attitudes and using problems of elderly people about VUI and voice robot. The questions include: “what do you think about the experience of talking with VUI or voice robot?,” “Did you have any problems when you talking with VUI or voice robot?,” “Did you always understand what the VUI or voice robot said? If not, what makes it incomprehensible?”

## 4. Results

### 4.1. Speech rate

The number of words per second during the subjects’ speech was defined as the subject’s speech rate, noted as *V_s_* in words/s (Li et al., 2019). Subjects’ raw recordings were imported into Adobe Audition 2020 for further processing. According to the speech rate test methodology proposed by Kim et al. (2015), subjects’ single complete speech was intercepted from the recording for speech rate analysis. If the subject paused for longer than 2 s in a single speech, the pause was deleted to obtain the corpus in the stable state, which was used to extract the subject’s speech rate. If the pause time was between 1 and 2 s, it was necessary to determine whether the subjects showed obvious doubt or nervousness according to the recorded video and choose the corpus that the subject was in a stable situation. If the subject paused for less than 1 s, it was considered normal.

The number of words is denoted as *S* and the total duration after deleting the silent pauses is denoted as *T* in single speech. The subjects’ speech rate *V_s_* during the voice interaction is calculated by Equation (1).


(1) $$V_{s}=\frac{S}{T}$$

**Figure 4:**
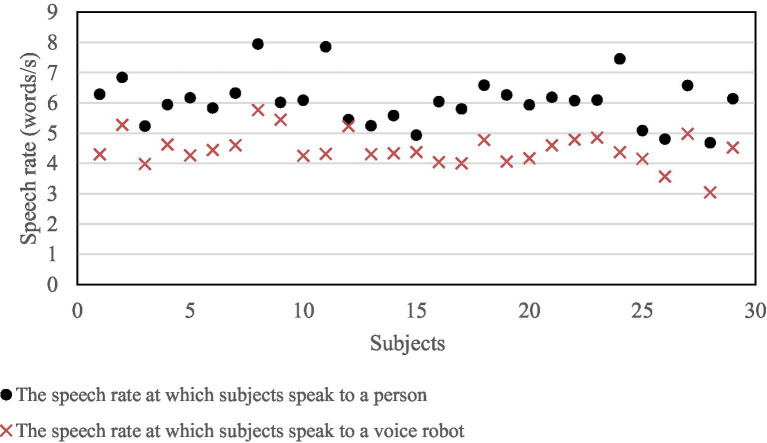
Distribution of subjects’ speech rate.

Before the experiment started, speeches subjects spoke to the experimenter were recorded. Six sentences were extracted to analyze the speech rates at which subjects speak to the experimenter. At the end of the experiment, six sets of speech rate data were collected from each subject separately. The mathematical expectation speech rates at which subjects speak to a person and a voice robot are shown in Figure 4. One subject did not complete the experiment, and then there were 29 subjects’ data. A paired *t*-test was performed on the two kinds of speech rates, and the results show a significant difference (*p* = 0.000), meaning subjects speak to a voice robot at a very different speech rate than a person.

### 4.2. Correlation between subjects’ speech rate and expected feedback speech rate

Experiments were conducted randomly with subjects on the three dialog tasks with six speech rates for feedback from the voice robot. After each session, subjects were asked to score the feedback speech rate on a satisfaction scale shown in Table 4. At the end of the experiment, the speech rate with the highest score was determined as the subjects expected feedback speech rate for this task. If more than one feedback speech rate configuration that got the highest score, then the mean of these configurations is defined as the expected feedback speech rate.

**Figure 5:**
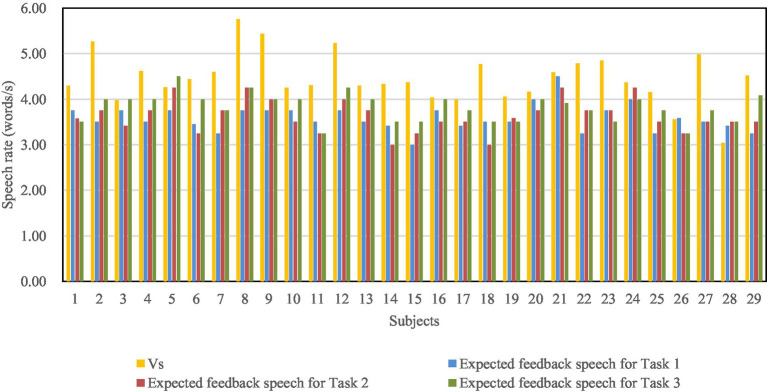
Distribution of subjects’ expected speech rate of feedback.

The subjects’ expected feedback speech rates of the three dialog tasks are, respectively, noted as *W*_1_, *W*_2_, and *W*_3_, which are shown in Figure 5. Paired *t*-test was performed on the speech rate at which subjects speak to a voice robot and the expected feedback speech rate. The results showed (Table 5) that there were significant differences (*p* = 0.00) between the two variables for all three dialog tasks. That means in all cases of dialog task type and feedback word count, subjects expected feedback speech rate lower than their speech rate at which they speak to a voice robot.

**Table 5:** Paired *t*-test of the subjects’ speech rate and the expected feedback speech rate.

	Mean							Std. deviation	Std. error mean	95% Confidence Interval of the Difference		*t*	df	Sig. (two-tailed)	Lower	Upper
Pair 1 *V_s_* – *W*_1_									0.872414	0.595469	0.110576	0.645910	1.098918	7.890	28	0.000
Pair 2 *V_s_* – *W*_2_									0.827931	0.517324	0.096065	0.631152	1.024711	8.618	28	0.000
Pair 3 *V_s_* – *W*_3_									0.641034	0.514465	0.095534	0.445343	0.836726	6.710	28	0.000

Pearson correlation analysis was conducted on the subjects’ speech rate *V_s_* and the subject’s expected system feedback speech rate *W*_1_, *W*_2_, *W*_3_, and the results are shown in Table 6. For Task 1, the results indicate no significant correlation (*r* = 0.113, *p* = 0.56) between the subjects’ speech rate and the expected feedback speech rate. For Task 2 and Task 3, there are significant positive correlations (*r* = 0.417, *p* = 0.025 and *r* = 0.399, *p* = 0.032) between the subjects’ speech rate and the expected feedback speech rate.

**Table 6:** Correlation between the subjects’ speech rate and the expected feedback speech rate.

		*V_s_*	*W*_1_	*W*_2_	*W*_3_
*V_s_*	Pearson correlation	1	0.113	0.417*	0.399^*^
	Sig. (two-tailed)		0.561	0.025	0.032
	*N*	29	29	29	29
*W*_1_	Pearson correlation	0.113	1		
	Sig. (two-tailed)	0.561			
	*N*	29	29		
*W*_2_	Pearson correlation	0.417*		1	
	Sig. (two-tailed)	0.025			
	*N*	29		29	
*W*_3_	Pearson correlation	0.399^*^	0.336		1
	Sig. (two-tailed)	0.032	0.075		
	*N*	29	29		29

**Figure 6:**
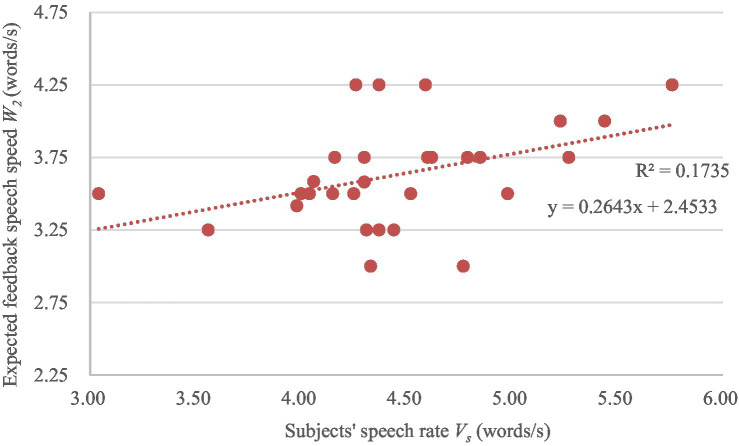
The variation of the expected feedback speech rate *W_2_* with the subjects’ speech rate V_s_.

**Figure 7:**
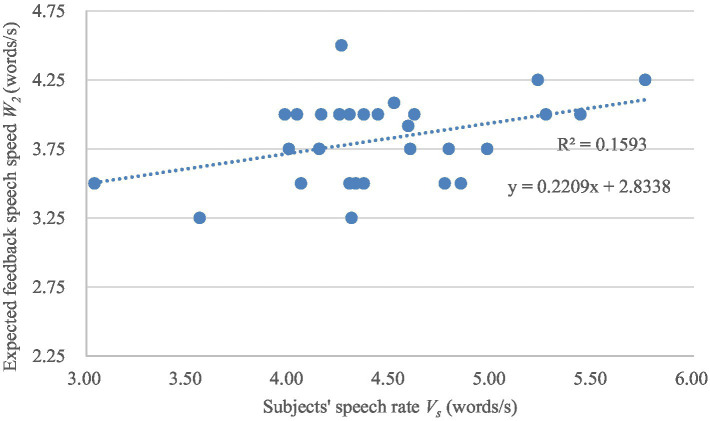
The variation of the expected feedback speech rate *W_3_* with the subjects’ speech rate V_s_.

The variations of the expected system feedback speech rate *W*_2_ and *W*_3_ with the subjects’ speech rate *V_s_* are shown in Figures 6, 7 separately. A linear regression analysis was conducted to analyze the specific performance of the correlation between subjects’ speech rates and the expected feedback speech rates. The linear regression models are also shown in Figures 6, 7. The *F*-test (*F* = 5.670, *p* = 0.025; *F* = 5.122, *p* = 0.032) and *T*-test (*t* = 2.381, *p* = 0.025; *t* = 2.263, *p* = 0.032) also show a significance of the linear relationship of the regression model and the regression coefficient.

### 4.3. The influence of dialog task on expected feedback speech rate

LSD-*t*-test was conducted on the subjects’ expected feedback speech rates *W*_1_, *W*_2_, and *W*_3_. The results are shown in Table 7. The significance of the chi-square test was *p* = 0.866, which validates the homogeneity of the collected data. As shown in Table 8, a one-way ANOVA was carried out on the subjects’ expected feedback speech rates in different types of dialog tasks. The results indicate no significant difference (*p* = 0.065) among the three expected feedback speech rates across dialog task types. That means dialog task type did not significantly affect the expected feedback speech rate.

**Table 7:** Homogeneity test of variance for *W*_1_, *W*_2_, and *W*_3_.

	Levin statistics		df 1	df 2	Sig.
Expected feedback speech rate *W*	Based on average	0.144	2	84	0.866
	Based on median	0.182	2	84	0.834
	Based on the median and with adjusted degrees of freedom	0.182	2	79.445	0.834
	Based on average after clipping	0.176	2	84	0.839

**Table 8:** One-way ANOVA test for expected feedback speech rate in different types of dialog tasks.

Expected feedback speech rate *W*					
	Sum of squares	df	Mean square	*F*	Sig.
Interblock	0.368	1	0.368	3.493	0.065
Interclass	8.950	85	0.105		
Total	9.318	85			

The *post hoc* LSD-*t*-test was conducted on the users’ expected system feedback speech rate *W*_1_, *W*_2_, and *W*_3_, and the correlation between the data was examined in two pairs. The results are depicted in Table 9. There is no significant difference between *W*_1_ and *W*_2_ (*p* = 0.831) under the conditions of different types of dialog tasks and the same number of words of feedback, which also indicates that the dialog task type does not affect the elderly’s satisfaction with the expected feedback speech rate.

**Table 9:** *Post hoc* LSD-*t* test for *W*_1_, *W*_2_, and *W*_3_.

(I) Group	(J) Group	Mean value (I-J)	Standard error	Sig.	95% confidence interval	
					Lower limit	Superior limit
*W_1_*	*W_2_*	−0.018621	0.087207	0.831	−0.19204	0.15480
	*W_3_*	−0.201207^*^	0.087207	0.024	−0.37463	−0.02779
*W_2_*	*W_1_*	0.018621	0.087207	0.831	−0.15480	0.19204
	*W_3_*	−0.182586^*^	0.087207	0.039	−0.35601	−0.00917
*W_3_*	*W_1_*	0.201207^*^	0.087207	0.024	0.02779	0.37463
	*W_2_*	0.182586^*^	0.087207	0.039	0.00917	0.35601

^*^ The significance level of the mean difference *p* < 0.05.

### 4.4. The influence of the feedback word count on expected feedback speech rate

The feedback word count of Task 1 and Task 2 was 37, and the mean value of the subjects’ expected feedback rate was 3.61 words/s and 3.63 words/s, respectively. The word count of Task 3 was 18, and the mean value of the expected feedback speech rate was 3.81 words/s.

As shown in Table 9, the subjects’ expected feedback speech rates *W*_1_ and*W*_3_ are significantly different (*p* = 0.024). *W*_2_ and *W*_3_ differ significantly (*p* = 0.039) alike. One-way ANOVA was also carried out on the subjects’ expected feedback speech rates in different word counts of dialog tasks. As shown in Table 10, the results indicate a significant difference (*p* = 0.005) among the three expected feedback speech rates across feedback word count. That means feedback word count affects the expected feedback speech rate significantly.

**Table 10:** One-way ANOVA test for expected feedback speech rate in different word counts of dialog task.

Expected feedback speech rate *W*					
	Sum of Squares	df	Mean square	*F*	Sig.
Interblock	0.846	1	0.846	8.484	0.005
Interclass	8.473	85	0.100		
Total	9.318	85			

It suggests that there was a difference in the expected speech rate of feedback between Task 1, “listen to the news,” and Task 3, “check the schedule.” Under conditions of different types of tasks and word counts of feedback, there is a difference in subjects’ expected feedback speech rates. Thus, it is essential to determine the factors affecting feedback speech rate. It indicates a difference between the subjects’ expected system feedback speech rate when completing the dialog task of acquiring information in Task 2 and Task 3, i.e., task scenarios with the same task type but different feedback word counts.

It demonstrates that the amount of system feedback words impacts users’ assessments of the system feedback speech rate. Subjects preferred a slower system feedback speech rate in the task scenario with more words of feedback than the feedback with fewer words.

Data analysis and post-experiment user interviews found that when the system feedback contained more words, the elderly’s expected feedback speech rate was significantly slower than the scenario with fewer words of feedback. It indicates that elderly people have limited cognitive ability when processing feedback information. When receiving more feedback, they take more time to remember and store the information; therefore, elderly people expect the feedback speech rate to be slower.

## 5. Discussion

There is a significant difference between the speech rate subjects speak to people and the voice robot. When elderly people speak to a robot, they consciously slow their speech rate. Most elderly people are not very familiar with voice interaction technology and the product, which led to a slight on voice robot (Branigan et al., 2011; Koulouri et al., 2016). “I think the robot may not be as smart as people, so I speak to it with a lower speech rate to ensure it hears me clearly and understand me,” subject No. 14 said in the interview after the experiment. Whether this phenomenon exists in people familiar with voice user interfaces and artificial intelligence technology is pending.

Elderly people expect the voice robot to give feedback at a slower speech rate than their own. From the aspect of language expression, speech rate reflects one’s cognitive, understanding and memory skills (Rönnberg et al., 1989; Sanju et al., 2019; Lotfi et al., 2020; Huiyang and Min, 2022). Elderly people want their interlocutor to talk to themselves with a lower speech rate to ensure they can hear and understand the speaker clearly. Even the speaker is a robot. Meanwhile, elderly people with faster speech rates expect a faster feedback speech rate, which confirms the current study results that people with faster speech rates expect their interlocutor to respond with a faster speech rate (Brown et al., 1985; Hargrave et al., 1994; Freud et al., 2018). These suggest to some extent that the speech convergence is applicable to the interaction between elderly people and VUI.

Compared with non-goal-oriented dialog, goal-oriented dialog features specific information acquisition, which may require the listener to concentrate more on the speaker’s feedback (Galley, 2007; Zhang et al., 2018; Stigall et al., 2020). Nevertheless, the results show that dialog task type did not significantly affect the expected feedback speech rate. Subject No. 7 said, “regardless of whether I have a clear goal of information acquisition, I always hope to hear the voice clearly and try my best to understand what I heard.” Based on our observations of the subjects during the experiments, they always try their best to listen and remember the voice robot’s feedback, even though they are not required to do so. This may be slightly different from the scenario of the elderly listening to the radio and music in their leisure time. Nearly 83% of the subjects (*N* = 25) said that they did not and would not remember all details of the music and radio they listened to in their leisure time.

As mentioned above, subjects always try their best to remember the information of the feedback from the voice robot. It is reasonable that feedback word count significantly affects the expected feedback speech rate. Although the experimental scenario is different from the real scenario of voice user interface usage, we still believe that the voice user interface designers should set a reasonable feedback speech rate to ensure users can accurately capture all the content of the feedback.

## 6. Conclusion

This paper focuses on the effects of the elderly’s speech rate, types of voice interaction task, and the word count of feedback on elderly people’s expected feedback speech rate. It is found that the elderly’s speech rate and the word count in a single feedback have a significant influence on elderly people’s expected feedback speech rate. However, dialog task type affects the expected feedback speech rate inapparently. The faster elderly people speak, the faster feedback speech rate they desire, but not faster than their own. The more words of feedback are, the slower the elderly’s expected feedback speech rate is. These results also provide valuable implications for VUI user experience design. The feedback speech rate should be defined according to the interacting speech rate of elderly people and the word count of feedback content.

This study was designed for theoretical and practical application, especially the linear regression models of subjects’ speech rate and their expected feedback speech rate could be applied to developing a voice robot or other applications with the voice user interface. Besides, the word count of the feedback is another factor that should be considered when defining the feedback speech rate. In this study, two typical scenarios, which contain 18 and 37 Chinese words, are used in the experiment, respectively. The results show a significant difference in the expected feedback speech rates. However, these two numbers are not guidelines to follow. More research should focus on the effect of word count or information blocks on the expected feedback speech rate.

This study is carried out with Chinese people, and the materials are also made of Chinese characters and mandarin, so the results just explain the interaction between Chinese elderly people and voice robots. As different languages are spoken with different speech rates intrinsically, the problems this paper focuses on could be studied further in other languages.

## Data availability statement

The original contributions presented in the study are included in the article/Supplementary material, further inquiries can be directed to the corresponding author.

## Ethics statement

The studies involving human participants were reviewed and approved by Ethics Committee of Shenzhen Technology University. The patients/participants provided their written informed consent to participate in this study.

## Author contributions

JW: conceptualization, methodology, investigation, supervision, project administration, and writing – review and editing. SY and ZX: data curation. SY and JW: formal analysis. SY: visualization. ZX: materials. JW and SY: writing – original draft preparation. All authors have read and agreed to the published version of the manuscript.

## Funding

This research was supported by the Humanities and Social Science Projects of the Ministry of Education of China (Grant No. 21YJC760078) and the Postgraduate Education and Teaching Reform Project of Guangdong Province of China (Grant No. 2022JGXM094).

## Conflict of interest

The authors declare that the research was conducted in the absence of any commercial or financial relationships that could be construed as a potential conflict of interest.

## Publisher’s note

All claims expressed in this article are solely those of the authors and do not necessarily represent those of their affiliated organizations, or those of the publisher, the editors and the reviewers. Any product that may be evaluated in this article, or claim that may be made by its manufacturer, is not guaranteed or endorsed by the publisher.
